# Inhibitory Effect of *Opuntia humifusa* Fruit Water Extract on Solar Ultraviolet-Induced MMP-1 Expression

**DOI:** 10.3390/ijms19092503

**Published:** 2018-08-24

**Authors:** Ah-Ram Han, Tae-Gyu Lim, Young-Ran Song, Mi Jang, Young Kyoung Rhee, Hee-Do Hong, Mi-Hyun Kim, Hyun-Jin Kim, Chang-Won Cho

**Affiliations:** 1 Traditional Food Research Group, Korea Food Research Institute, Wanju-gun 55365, Jeollabuk-do, Korea; Han.Ah-ram@kfri.re.kr (A.-R.H.); tglim@kfri.re.kr (T.-G.L.); Song.Young-ran@kfri.re.kr (Y.-R.S.); jangmi@kfri.re.kr (M.J.); ykrhee@kfri.re.kr (Y.K.R.); honghd@kfri.re.kr (H.-D.H.); Kim.Mi-hyun@kfri.re.kr (M.-H.K.); 2 Department of Food Science and Technology, Division of Applied Life Sciences (BK21 plus), and Institute of Agriculture and Life Science, Gyeongsang National University, Jinju-si 52828, Gyeongsangnam-do, Korea; hyunjkim@gnu.ac.kr

**Keywords:** *Opuntia humifusa*, sUV damage, ROS, MMP-1, photoaging

## Abstract

*Opuntia humifusa* is a type of cactus whose fruits have been used in folk medicine for the treatment of several diseases. In the present study, we aimed to determine whether *O. humifusa* fruit water extract (OHE) has inhibitory effects against solar ultraviolet (sUV)-induced matrix metalloproteinase-1 (MMP-1) expression. In ex vivo human skin, we found that OHE suppressed sUV radiation-induced MMP-1 expression. The inhibitory effect of OHE was confirmed in human dermal fibroblasts. OHE treatment reduced sUV-induced MMP-1 expression by suppressing reactive oxygen species (ROS) generation and phosphorylation of c-Jun, a component of transcription factor activator protein 1 (AP-1). On the other hand, OHE recovered the tissue inhibitor of matrix metalloproteinase 1 (TIMP-1) and type 1 collagen production attenuated by sUV. As upstream signaling pathways for AP-1, MKK4-JNK, MEK-ERK, and MKK3/6-p38 phosphorylation were downregulated by OHE treatment. In addition, OHE exhibited DPPH radical scavenging activity. These findings demonstrate that OHE has a preventive effect against sUV-induced skin damage via suppression of pathways triggered by ROS.

## 1. Introduction

Human skin is in direct contact with the environment and therefore undergoes aging as a consequence of environmental damage. The primary environmental factor that causes human skin aging is ultraviolet (UV) radiation, and repeated exposure to UV causes premature skin aging (photoaging) [1,2]. Photoaging is characterized by morphological changes that include deep wrinkles, loss of elasticity, dryness, and laxity and histological changes such as connective tissue alterations [2]. UV exposure induces the generation of reactive oxygen species (ROS), which can damage lipids, proteins, and deoxyribonucleic acid (DNA) and promote apoptosis [3]. ROS are also involved in physiological processes such as cell signaling, proliferation, and tumor suppression, leading to oxidative stress after the depletion of cellular defense mechanisms [4,5].

UV radiation-induced ROS production, in turn, activates the mitogen-activated protein kinase (MAPK) signaling cascades, which consist of extracellular signal-regulated kinases (ERK), p38 MAPK, and c-Jun N-terminal kinases (JNK) that induce subsequent activation of transcription factor activator protein 1 (AP-1) [6]. A subunit of AP-1, c-Jun is stimulated by UV radiation in response to JNK activation [7]. The increased activation of AP-1 is involved in the degradation of collagen via inducing matrix metalloproteinase (MMP) production, which leads to alteration of the extracellular matrix (ECM) in skin [8]. MMP-1 plays an important role in the process of photoaging because it specifically cleaves the type 1 collagen, which is the most abundant protein in skin connective tissue [8,9].

*Opuntia humifusa* is a member of the Cactaceae family and widely distributed in semi-arid countries throughout the world [10]. In Korea, *O. humifusa* has been cultivated in inland areas. *O. humifusa* contains high concentrations of polyphenols, flavonoids, and dietary fibers [11,12,13]. It has been reported that extracts of *O. humifusa* exhibit anti-inflammatory [14], anti-diabetic [15], and antioxidant effects [13,14]. Recently, extracts from *O. humifusa* have been reported to exert anticancer effects on human HeLa cervical cancer cells [16]. Other recent studies described the potential of *O. humifusa* cladode extract to be used in skincare; the extract suppressed transepidermal water loss and erythema formation by regulating UVB-induced hyaluronic acid (HA) production [17]. Furthermore, *O. humifusa* fruit extract was suggested for use as a skin-whitening agent as it inhibited tyrosinase and melanogenesis associated transcription factor (MITF) activity. However, the effect of *O. humifusa* fruit water extract (OHE) on skin aging is unclear.

In the present study, we investigated whether OHE can promote an anti-photoaging effect in sUV radiated primary human dermal fibroblasts (HDFs) by analyzing the expression of MMP-1 and collagen, and the underlying signal pathway(s) involved. The ex vivo effect of OHE was also assessed using sUV-radiated human skin model.

## 2. Results

### 2.1. Analysis of Major Phytochemicals in OHE

**Figure 1:**
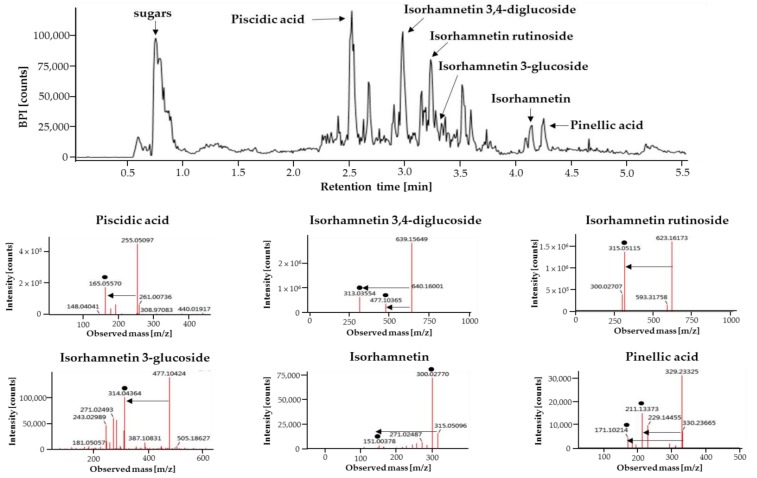
The liquid chromatogram of *Opuntia humifusa* fruit water extract (OHE) and tandem mass spectrometry (MS/MS) data of major phytochemicals. Phytochemicals in the OHE were analyzed using UPLC-Q-TOF MS equipped with an Acquity UPLC BEH C18 column (2.1 mm × 100 mm, 1.7 µm) and negative mode ESI. Major phytochemicals were identified by using the UNIFI scientific information system with various LC/MS online databases. Solid spots indicated mass fragments of major phytochemicals.

Phytochemicals in OHE were analyzed using ultra-performance liquid chromatography–quadrupole-time of flight (UPLC-Q-TOF) mass spectrometry (MS) operating in an electrospray ionization (ESI) negative mode are shown in Figure 1. A total of six compounds were tentatively identified by comparison of their retention time, precise molecular mass, and the patterns of MS fragmentations. Sugars, piscidic acid, isorhamnetin 3,4-diglucoside, and isorhamnetin rutionside were identified as the major phytochemicals in OHE. Moreover, isorhamnetin, isorhamnetin 3-glucoside, and pinellic acid were identified.

### 2.2. Effects of OHE on Cell Viability and Antioxidative Activity 

**Figure 2:**
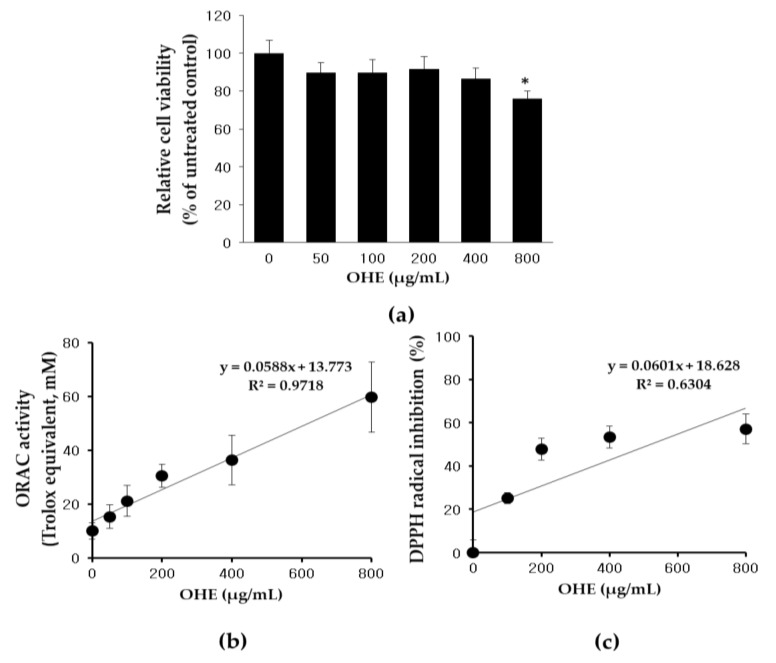
The effects of *Opuntia humifusa* fruit water extract (OHE) on cell viability (**a**), Regression coefficient graph of the OHE on oxygen radical absorbance capacity (ORAC) (**b**), and DPPH radical-scavenging activity (**c**). Data are presented as the mean ± standard deviation (SD) and analyzed by oneway ANOVA followed by Dunnett’s test. * *p* < 0.05 vs. control group.

A 3-(4,5-dimethylthiazol-2-yl)-5-(3-carboxymethoxyphenyl)-2-(4-sulfophenyl)-2H tetrazolium (MTS) assay was conducted to estimate the cytotoxic effect of OHE treatment. HDFs were treated with various concentrations of OHE for 24 h. OHE showed no cytotoxic effects on HDFs up to 400 μg/mL. However, the cell viability was significantly affected by OHE at 800 μg/mL (Figure 2a). An oxygen radical absorbance capacity (ORAC) assay was then used to determine the antioxidant capacity of OHE in vitro (Figure 2b), and the results demonstrated that OHE significantly increased the antioxidant capacity in a dose-dependent manner. Furthermore, we analyzed the radical-scavenging activity of OHE using a 2,2-diphenyl-1-picrylhydrazyl (DPPH) assay. As shown in Figure 2c, OHE inhibited DPPH radicals by 53.3% at 400 μg/mL dose-dependently.

### 2.3. Effects of OHE on Intracellular ROS Production in Primary HDFs 

**Figure 3:**
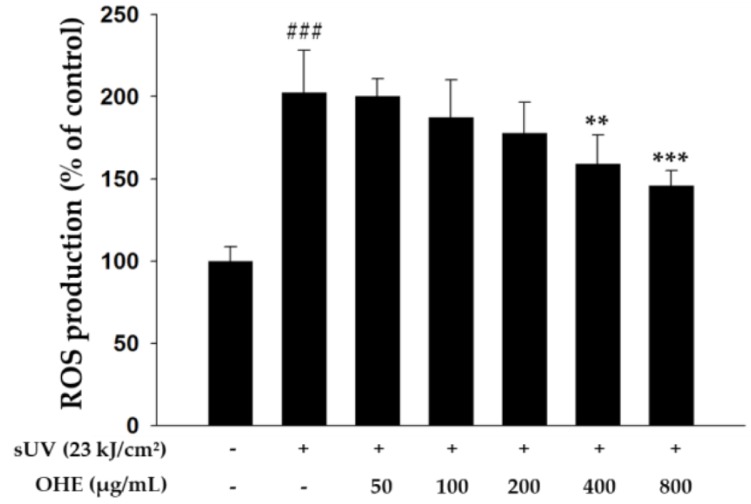
The inhibitory effect of *Opuntia humifusa* fruit water extract (OHE) on solar ultraviolet (sUV)-induced intracellular reactive oxygen species (ROS) production in human dermal fibroblasts (HDFs). Data are presented as the calculated percentage of the control group expression and shown as the mean ± standard deviation (SD). ^###^ *p* < 0.001 vs. control group (Student’s *t*-test), ** *p* < 0.01, *** *p* < 0.001 vs. UV-radiated groups (Dunnett’s test).

ROS generated by UV radiation promotes MMP expression, leading to the degradation of collagen and ECM proteins [8]. Therefore, inhibition of ROS production may be an effective strategy to alleviate UV-induced skin damage or photoaging. Thus, we investigated the effects of OHE treatment on sUV radiation-induced ROS generation in HDFs. As shown in Figure 3, sUV-radiated cells exhibited markedly increased ROS generation compared with non-irradiated cells, while treatment with higher concentrations of OHE (200, 400, and 800 μg/mL) significantly decreased the ROS production compared with that in sUV-radiated control cells. These results indicated that OHE may have an inhibitory effect on the production of intracellular ROS under sUV exposure. 

### 2.4. Effects of OHE on sUV-Induced Photoaging in Human Skin 

**Figure 4:**
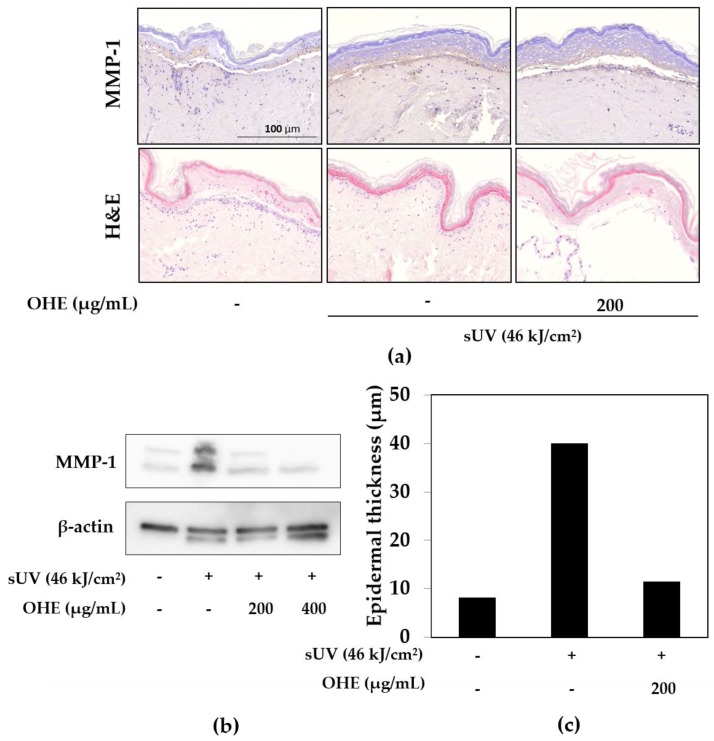
The effect of *Opuntia humifusa* fruit water extract (OHE) on solar ultraviolet (sUV)-radiated ex vivo human skin. (**a**) Histopathological analysis of matrix metalloproteinase-1 (MMP-1) and hematoxylin and eosin (H&E)-stained sections in human skin. (**b**) MMP-1 protein levels in human skin. MMP-1 expression was measured using western blot analysis, as described in Materials and Methods. (**c**) Qualitative data of epidermal thickness of human skin.

MMP-1, also known as fibroblast collagenase, plays a critical role in UV-mediated photoaging leading to ECM degradation in the skin [18]. We hypothesized that suppressing the UV-induced MMP-1 expression could prevent skin photoaging. To determine the effect of sUV exposure and OHE treatment on MMP-1 expression, ex vivo human skin was used. MMP-1 expression was noticeably increased by sUV exposure (Figure 4a), while treatment with OHE suppressed the sUV-induced MMP-1 expression. We also implemented western blot analysis using a skin tissue lysate, which showed similar results. The sUV-radiated group exhibited an increase in MMP-1 expression, and OHE pretreatment significantly reduced this increase (Figure 4b). Histological studies showed that enhanced epidermal thickness induced by UV radiation could be used as a parameter of skin photoaging [19]. To determine the effect of sUV exposure on epidermal thickness, human skin was stained with hematoxylin and eosin (H&E). As shown in Figure 4a,c, the epidermal thickness was substantially increased by sUV exposure compared with that in the normal skin. Surprisingly, OHE treatment dramatically reduced the epidermal thickness of the human skin.

### 2.5. Effects of OHE on MMP-1, Type 1 Collagen, and Tissue Inhibitor of Matrix Metalloproteinase 1 (TIMP-1) Expression in sUV-Exposed Primary HDFs 

**Figure 5:**
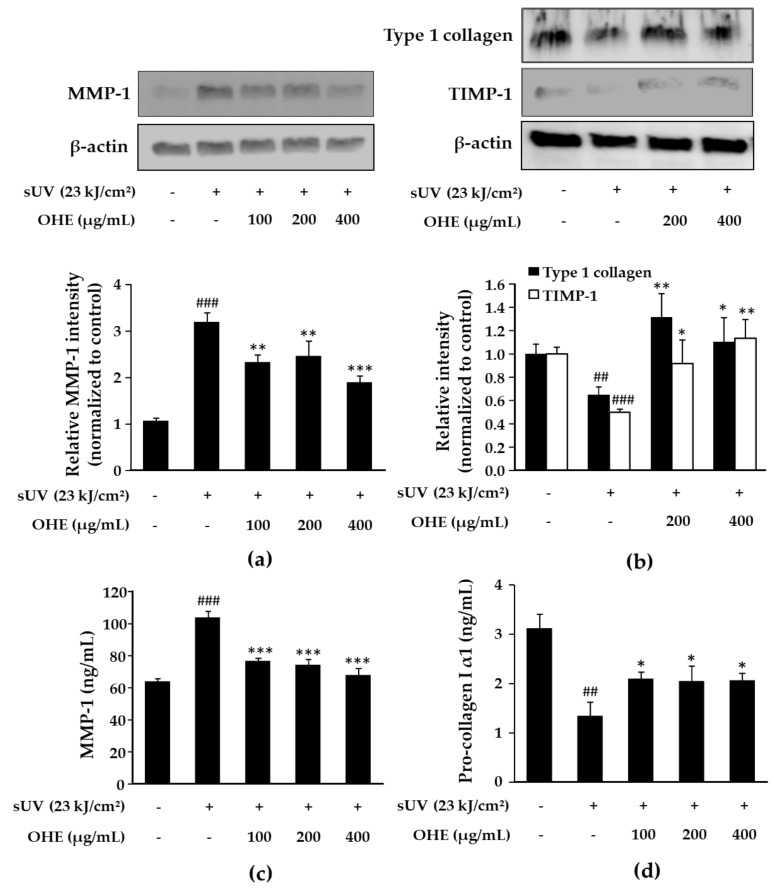
The inhibitory effect of *Opuntia humifusa* fruit water extract (OHE) on solar ultraviolet (sUV)-induced MMP-1, type 1 collagen, and TIMP-1 expression in human dermal fibroblasts (HDFs). HDFs were treated with OHE for 1 h, followed by radiation with sUV and incubation for 48 h. (**a**) MMP-1 expression levels, (**b**) Type 1 collagen and TIMP-1 expression levels were measured by western blot. (**c**) MMP-1 secretion levels, (**d**) Procollagen 1 secretion levels in the culture media were measured by enzyme-linked immunosorbent assay (ELISA). Data are presented as the mean ± standard deviation (SD). ^##^ *p* < 0.01, ^###^ *p* < 0.001 vs. control group (Student’s *t*-test), * *p* < 0.05, ** *p* < 0.01, *** *p* < 0.001 vs. UV-radiated groups (Dunnett’s test).

We explored the effects of OHE treatment on sUV-induced MMP-1 expression in HDFs. Similar to the results obtained using ex vivo human skin (Figure 4b), OHE downregulated the sUV radiation-induced MMP-1 protein expression (Figure 5a) and secretion (Figure 5c). On the other hand, OHE upregulated the expression of TIMP-1, an inhibitor of MMP-1 [20] (Figure 5b). Degradation of the connective tissue ECM, which mainly comprises collagen and elastin, is regulated by several inducible MMPs and TIMP-1 [21]. Thus, we further examined the effects of OHE on the sUV-induced expression of the type 1 collagen. As shown in Figure 5b,d, sUV radiation reduced the level of the type 1 collagen compared with that in the control group, and OHE inhibited this downregulation. These results suggested that OHE inhibited sUV-induced collagen degradation by regulating the MMP-1 and TIMP-1 expression.

### 2.6. OHE Suppresses sUV-Induced MKK4/JNK/c-Jun, MEK/ERK, and MKK3/6/p38 Signaling Pathways 

**Figure 6:**
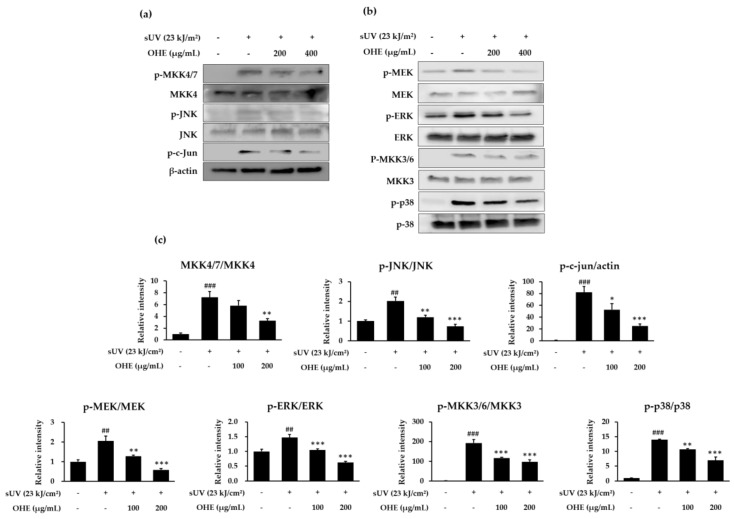
The inhibitory effect of the *Opuntia humifusa* fruit water extract (OHE) on solar ultraviolet (sUV)-induced phosphorylation of Mitogen-activated protein kinases (MAPKs) and MAPK kinases (MAPKKs) in human dermal fibroblasts (HDFs). HDFs were treated with OHE for 1 h following treatment with sUV radiation. (**a**,**b**) Phosphorylation and expression were detected by western blot assay, and (**c**) relative expression levels were calculated using the Image J software version 1.8.0_66 (National Institutes of Health, Bethesda, MD, USA). Data are presented as the mean ± standard deviation (SD). ^##^ *p* < 0.01, ^###^ *p* < 0.001 vs. control group (Student’s *t*-test), * *p* < 0.05, ** *p* < 0.01, *** *p* < 0.001 vs. UV-radiated groups (Dunnett’s test).

It is well known that the JNK, ERK, and p38 signaling pathways modulate UV-induced MMP-1 expression via AP-1 activation [22]. Therefore, we investigated which signaling pathways were involved in the reduction of sUV radiation-induced MMP-1 expression by OHE. As seen in Figure 6, phosphorylation levels of MAPKs and MAPKKs were remarkably increased by sUV radiation, and OHE suppressed the sUV-induced phosphorylation of MKK4/JNK, MEK/ERK, and MKK3/6/p38. Moreover, the levels of phosphorylated c-Jun (a subunit of AP-1) were increased in sUV-radiated HDFs and suppressed by OHE treatment (Figure 6c). Collectively, these results supported the notion that OHE suppresses the sUV-induced MMP-1 expression by inhibiting the MAPKK and MAPK signaling pathways. 

## 3. Discussion

Photoaging results from the accumulated damage of the skin caused by chronic UV exposure, depending mainly on the intensity of sun exposure and skin pigment [2,23]. This UV-induced damage is characterized by increased ROS generation, which induces oxidative damage to the structural dermal proteins such as collagen and elastin. UV exposure-induced ROS stimulate expression of MMP-1, which causes collagen degradation, resulting in the damage to dermal connective tissues and the formation of wrinkles, visible signs of skin aging [5]. Therefore, the inhibition of ROS production is suggested as an important target for anti-photoaging through the suppression of MMP-1 production. In recent years, many natural skincare cosmetics have been developed to treat skin aging, inhibiting MMPs and stimulating the synthesis of collagen in addition to antioxidant properties [24].

In the present study, OHE inhibited sUV-induced expression of MMPs and recovered TIMP-1 and type 1 collagen expression by downregulating AP-1 phosphorylation and MAPKK/MAPK signaling pathways in HDFs. This anti-aging effect of OHE appeared to be due to its ability to reduce the oxidative stress induced by sUV via inhibition of intracellular ROS generation. To prove that anti-photoaging effect of OHE was not caused by the sunscreen effect, we tested the free media and 400 μg/mL of OHE, and obtained UV absorbance spectra, expressing the relationship between absorbance and wavelength in the range from 280 to 400 nm. The results showed that the absorbance values according to the wavelength of free media and 400 μg/mL of OHE were almost matched, so we concluded that OHE did not absorb the sUV (Supplementary Figure S1).

We further confirmed that OHE has a protective effect on skin damaged by sUV radiation in ex vivo human skin. Treatment with OHE attenuated sUV radiation-induced MMP-1 expression and reversed sUV-induced skin thickening. In regards to the reversal of sUV-induced skin thickening by OHE, we could not have tested the H&E staining of skin tissue including negative control (OHE treatment without UV-radiation) to exclude the toxicity of OHE on skin tissue. However, we tested it with no cell cytotoxicity in the sample concentration range by MTS assay in Figure 2a, so we suppose that OHE would not be dangerous for the epidermal barrier.

*O. humifusa* originated in southern Canada and the eastern United States and is widely distributed in the southern regions of the Korean peninsula, surviving Korean winters even in areas where the temperatures reach −24 °C because of their highly efficient usage of water [25]. Since ancient times, *O. humifusa* fruits have been used in folk medicine for the treatment of several diseases such as asthma, stomach ulcers, and indigestion [26]. The fruits are also used to prepare value-added food products, such as jams, sweets, ice cream, and alcoholic and non-alcoholic beverages [27]. The *O. humifusa* fruit is rich in nutrients such as betalains, polysaccharides, vitamins, minerals, and dietary fiber, containing high concentrations of phenolic acids and flavonoids, which have been identified as antioxidants [13,15]. 

There are many reports on the phytochemical components of *Opuntia* spp., consisting of phenolic compounds known to have antioxidant properties, in particular, flavonoids such as isorhamnetins (namely 3-*O*-glycoside derivatives), kaempferol, quercetin, taxifolin, and phenolic acids such as ferulic, piscidic, and eucomic acids [13,28,29]. Similarly, OHE analyzed by liquid chromatography–tandem MS (LC/MS/MS) showed the presence of piscidic acid, pinellic acid, and flavonoids, namely, isorhamnetins including isorhamnetin 3,4-diglucoside and isorhamnetin rutinoside, and isorhamnetin-3 glucoside (Figure 1). Piscidic acid belongs to the family of phenylpyruvic acid derivatives and is described as a strong chelator of iron with antioxidant properties [30]. Previous study showed that isorhamnetin has direct antioxidative and superoxide anion generation-suppressing activities [31]. Onions treated by high-pressure processing increased various flavonoids extractability including isorhamnetin 3,4-diglucoside, and increased anti-inflammatory and antioxidant activities [32]. Isorhamnetin rutinoside was reported to have a significant antioxidant and anti-inflammatory effect on human peripheral blood mononuclear cells stimulated with phytohaemagglutinin [33]. In addition, isorhamnetin 3-glucoside, isolated from *Cochlospermum religiosum*, was reported to significantly retard selenite-induced cataract in vitro by virtue of its antioxidant properties [34]. Thus, the accumulated evidence indicates that the antioxidant capacity of OHE may be attributed to its flavonoid and phenolic acid constituents. Further studies are required to quantitatively analyze OHE and identify the compounds responsible for the anti-photoaging effects.

## 4. Materials and Methods

### 4.1. Reagents

The MTS solution was purchased from Promega (Madison, WI, USA). Dulbecco’s modified Eagle medium (DMEM), fetal bovine serum (FBS), penicillin/streptomycin, and MMP-1 antibody were procured from Thermo Fisher Scientific (San Jose, CA, USA). Primary antibodies recognizing phosphorylated MEK (Ser^217/221^), total MEK, phosphorylated SEK1/MKK4 (MKK4, Ser^257^/Thr^261^), phosphorylated MKK3/6 (Ser^189^/Ser^207^), phosphorylated p44/p42 MAPK (ERK1/2) (Thr^202^/Tyr^204^), phosphorylated SAPK/JNK (Thr^183^/Tyr^185^), total MKK3, phosphorylated c-Jun (Ser^73^), phosphorylated p38 (Tyr^180/182^), and total p38 were purchased from Cell Signaling Technology (Danvers, MA, USA). Antibodies against total ERK, total MKK4, and total JNK were obtained from Santa Cruz Biotechnology (Santa Cruz, CA, USA). 

### 4.2. Sample Preparation and Extraction Procedure

Fruits of *O. humifusa* grown in the Asan area (Chungcheongnam-do, Korea) were harvested in October 2016, washed with water, air dried, cut in half, and then freeze-dried. The samples were ground with a blender (Wonder Blender, OSAKA CHEMICAL Co., Osaka, Japan) to obtain a fine powder. Ten grams of dried powder was extracted using an Ultrasonic Processor VCX 750 (Sonics & Materials, Inc., Newtown, CT, USA) with 1000 mL of distilled water for 30 min. The extract was centrifuged at 6000 rpm for 20 min, and then the supernatants were freeze-dried. The freeze-dried OHE was stored at −20 °C until further use, in which the yield of OHE was 31.7% of the dried weight of *O. humifusa* fruits.

### 4.3. Analysis of Phytochemicals in OHE 

Phytochemicals in the OHE were analyzed using UPLC-Q-TOF MS (Waters, Milford, MA, USA). The OHE was injected into an Acquity UPLC BEH C18 column (2.1 mm × 100 mm, 1.7 µm; Waters) at a column temperature of 40 °C. The mobile phase consisted of water with 0.1% formic acid (FA) (A) and acetonitrile with 0.1% FA (B) at a flow rate of 0.35 mL/min for 10 min. The eluents were analyzed using a Q-TOF mass spectrometer with negative mode ESI. The scan range of TOF MS data was from 50 to 1500 *m*/*z* with a scan time of 0.2 s. The capillary voltage was set at 2.5 kV for the negative mode, while the sample cone voltage was 40 V. The desolvation flow rate was 900 L/h at a temperature of 400 °C, and the source temperature was set to 100 °C. Leucine-enkephalin ([M + H] = *m*/*z* 556.2771; [M − H] = *m*/*z* 554.2615) was used as a reference for lock mass at a frequency of 10 s. The MS/MS spectra were obtained using collision energy ramps from 20 to 45 eV. Major phytochemicals were identified by using the UNIFI scientific information system (Waters) with various LC/MS online databases.

### 4.4. Cell Culture

Primary HDFs were kindly provided by Jin Ho Chung (College of Medicine, Seoul National University, Seoul, Korea). The HDFs were cultured in monolayers with DMEM containing 10% FBS and 2 mM L-glutamine at 37 °C in a humidified atmosphere containing 5% CO_2_. 

### 4.5. sUV Light Exposure 

HDFs were exposed to sUV radiation at 23 kJ/cm^2^ in serum-free media. Human skin-equivalent samples were exposed to sUV radiation at 46 kJ/cm^2^ in serum-free media. The sUV source was purchased from Q-Lab Corporation (Cleveland, OH, USA). The UVA-340 lamps were used to produce an optimal simulation of sunlight in the critical short wavelength region from 365 nm down to the solar cutoff of 295 nm with a peak emission of 340 nm. The percentage of UVA and UVB produced by the UVA-340 lamps was measured with a UV meter at 94.5% and 5.5%, respectively [35].

### 4.6. Cell Viability Assay

Cell cytotoxicity was measured using an MTS assay. HDFs were cultured in 96-well plates at a density of 5 × 10^4^ cells/well and incubated in DMEM containing 10% FBS and 1% penicillin/streptomycin at 37 °C with 5% CO_2_. Cells were starved in serum-free DMEM overnight and treated with OHE at the indicated concentrations for 24 h. Cells were then treated with 20 µL of MTS solution activated with phenazine methosulfate (PMS) for 1 h. The absorbance at 490 nm was measured using a microplate reader (Infinite 200 PRO NanoQuant, Tecan, Männedorf, Switzerland). 

### 4.7. Measurement of MMP-1 and Type 1 Procollagen Production

The cultured HDFs were starved in serum-free DMEM for 24 h and treated with 100, 200, or 400 μg/mL of OHE in serum-free media for 1 h, followed by sUV (23 kJ/cm^2^) radiation. After 48 h, the supernatants were collected and analyzed with Human Total MMP-1 DuoSet and Human Pro-collagen I alpha 1 DuoSet ELISA kits (R&D Systems, Inc., Minneapolis, MN, USA), according to the manufacturer’s protocols. 

### 4.8. Western Blot

After serum starvation overnight, the cells were exposed to various concentrations of OHE for 1 h before exposure to sUV radiation. The cells were disrupted with 1× Cell Lysis Buffer (Cell Signaling Technology) before the protein concentration was measured using a Pierce™ BCA Protein Assay Kit (Thermo Fisher Scientific). Protein samples were loaded into a 10% sodium dodecyl sulfate (SDS)-polyacrylamide gel (Bio-Rad, Hercules, CA, USA) for electrophoresis before transfer to an Immobilon P membrane (Millipore, Billerica, MA, USA). Non-specific binding was blocked with 5% fat-free milk for 1 h, and the membrane was incubated with the specific primary antibody at 4 °C overnight. Protein bands were detected with a chemiluminescence detection kit (GE Healthcare, Rahway, NJ, USA) after hybridization with a horseradish peroxidase (HRP)-conjugated secondary antibody (Cell Signaling Technology).

### 4.9. DPPH Radical Scavenging Assay

OHE (or ethanol itself as a control) was diluted to final concentrations of 800, 400, 200, 100, and 50 μg/mL in ethanol. One hundred microliters of 700 μM DPPH–ethanol solution was added to 100 μL of the sample solution placed in a 96-well microplate at room temperature in the dark. After 30 min of incubation, the absorbance was measured at 517 nm with a microplate reader (Infinite 200 PRO NanoQuant). Ascorbic acid was used as a positive control. The scavenging activity was determined by the following equation:

$$\%\;\mathrm{scavenging activity}=\left[1-\left(\frac{A_{sample}}{A_{control}}\right)\right]\times 100$$

### 4.10. Measurement of Intracellular ROS Production

Cells were treated with OHE for 3 h in FBS-free medium and then washed with phosphate-buffered saline (PBS). After washing, cells were treated with 25 μM of 2′,7′-dichlorofluorescein diacetate (DCFDA; Molecular Probes Inc., Eugene, OR, USA) and incubated for 40 min at 37 °C in a CO_2_ incubator. Following radiation with 23 kJ/cm^2^ of sUV, the ROS production was measured using a fluorescence microplate reader (SpectraMax M2e, Molecular Devices, San Jose, CA, USA) at an excitation of 485 nm and emission of 535 nm.

### 4.11. ORAC Assay

The measurement of ORAC was performed following previously described procedures [36]. In brief, a 75 mM phosphate buffer (pH 7.4) was prepared for the generation of 40 nM fluorescein and 150 mM 2,20-azobis-(2-amidinopropane) dihydrochloride (AAPH) solutions. The blank well received 25 μL of distilled water, the sample wells received 25 μL of OHE (solutions at different concentrations), while the standard wells were filled with 25 μL of trolox standard solutions prepared at different concentrations. Then, each well received 150 μL of fluorescein, followed by incubation at 37 °C for 45 min. The total volume of each well was brought to 200 μL by adding an AAPH solution. The fluorescence was recorded every minute for 60 min using a fluorescence spectrophotometer (Infinite NanoQuant M200, Tecan, Zürich, Switzerland) with an excitation wavelength of 485 nm and emission wavelength of 520 nm. The data analysis was subsequently performed by calculating the differences in net area under the fluorescein decay curve (AUC) between the blank and sample. The resultant values were expressed as μmol trolox equivalents (TE).

### 4.12. Human Skin Sample Preparation 

The abdominal skin was donated by a female Caucasian patient (Biopredic International, Saint Grégoire, France). The skin was maintained in a skin culture medium (Biopredic International) containing 10% FBS (*v*/*v*) at 37 °C in a 5% CO_2_ incubator, with the experiment carried out over 8 days. The tissue was pretreated with OHE for 1 h before radiation with 46 kJ/cm^2^ of sUV each day. The media were changed every 2 days. At the conclusion of the experiments, half of the tissue was immunohistochemically analyzed, and the other half of the tissue was lysed with 1× Cell Lysis Buffer (Cell Signaling Technology, Danvers, MA, USA) with homogenization. The protein expression was evaluated by western blot analysis.

### 4.13. Immunohistochemical Staining

Immunohistochemical staining was performed by ABION CRO (Seoul, Korea). The tissue was fixed in 10% formalin solution, dehydrated through a graded ethanol series, cleared in xylene, and processed for embedding in paraffin wax, according to routine protocols. The sections were incubated in a solution of 0.3% H_2_O_2_ for 15 min to inhibit endogenous peroxidase activity. Sections were then incubated for 1 h at room temperature with primary antibodies against MMP-1 (MAB901, R&D Systems, Inc.) diluted at a ratio of 1:200. An EnVision detection system using anti-mouse antibodies (K4001, DAKO, Glostrup, Denmark) was used according to the manufacturer’s instructions. Tissue sections were stained with liquid diaminobenzidine tetrahydrochloride (DAB+), a high-sensitivity substrate–chromogen system (K3468, DAKO). Counterstaining was performed with Mayer’s H&E. The tissue sections were visualized with a light microscope (BX40, Olympus, Tokyo, Japan). Pannoramic Viewer ver. 1.15.4 (3DHISTECH Ltd., Budapest, Hungary) was used to measure epidermal thickness.

### 4.14. Statistical Analysis

The results are expressed as the mean and standard deviation (SD) of three independent experiments. All data were analyzed using the SPSS ver. 20.0 software (IBM, Armonk, NY, USA). Differences between the control and the solar UV-radiated control were assessed with a Student’s *t*-test. To compare difference among the solar UV-radiated groups, one-way analysis of variance (ANOVA) was used followed by Dunnett’s post hoc test. Statistical significance was assessed as *p* < 0.05 

## 5. Conclusions

In this study, our findings demonstrated that OHE treatment suppressed sUV-induced ROS generation and its downstream signaling pathways, such as MAPKKs/MAPKs and AP-1. Ultimately, we showed that OHE inhibited the sUV radiation-induced MMP-1 expression and collagen degradation in human skin fibroblasts and confirmed similar effects in human skin. These results indicated that antioxidant effects of OHE contribute to the suppression of sUV-induced photo-damage in human skin, suggesting its potential use in cosmetic products, particularly in anti-wrinkle products.

## Supplementary Materials

Supplementary materials can be found at http://www.mdpi.com/1422-0067/19/9/2503/s1.

## Abbreviations

AAPH	2,20-azobis-(2-amidinopropane) dihydrochloride
AP-1	Activator protein 1
DMEM	Dulbecco’s modified Eagle medium
DPPH	2,2-diphenyl-1-picrylhydrazyl
ECM	Extracellular matrix
ERK	Extracellular signal-regulated kinases
ESI	Electrospray ionization
FA	Formic acid
FBS	Fetal bovine serum
HDFs	Human dermal fibroblasts
H&E	Hematoxylin and eosin
JNK	c-Jun N-terminal kinases
LC	Liquid chromatography
MAPK	Mitogen-activated protein kinase
MAPKK	MAPK kinase
MMP-1	Matrix metalloproteinase-1
MS	Mass spectrometry
MS/MS	Tandem mass spectrometry
MTS	3-(4,5-dimethylthiazol-2-yl)-5-(3-carboxymethoxyphenyl)-2-(4-sulfophenyl)-2H tetrazolium
OHE	*Opuntia humifusa* fruit water extract
ORAC	Oxygen radical absorbance capacity
ROS	Reactive oxygen species
SD	Standard deviation
sUV	Solar ultraviolet
TIMP-1	Tissue inhibitor of matrix metalloproteinase 1
UPLC-Q-TOF	Ultra-performance liquid chromatography–quadrupole-time of flight
UV	Ultraviolet
