# Supplementary material for: Inhibitory Effect of Opuntia humifusa Fruit Water Extract on Solar Ultraviolet-Induced MMP-1 Expression
Source: Int J Mol Sci. 2018 Aug 24;19(9):2503. doi: 10.3390/ijms19092503 (PMC6163292; doi:10.3390/ijms19092503)
Supplement: Supplementary file 1 [file ijms-19-02503-s001.pdf]

Supplementary Materials: Inhibitory Effect of *Opuntia humifusa* Fruit Water Extract on Solar Ultraviolet-Induced MMP-1 Expression

Ah-Ram Han, Tae-Gyu Lim, Young-Ran Song, Mi Jang, Young Kyoung Rhee, Hee-Do Hong, Mi-Hyun Kim, Hyun-Jin Kim and Chang-Won Cho

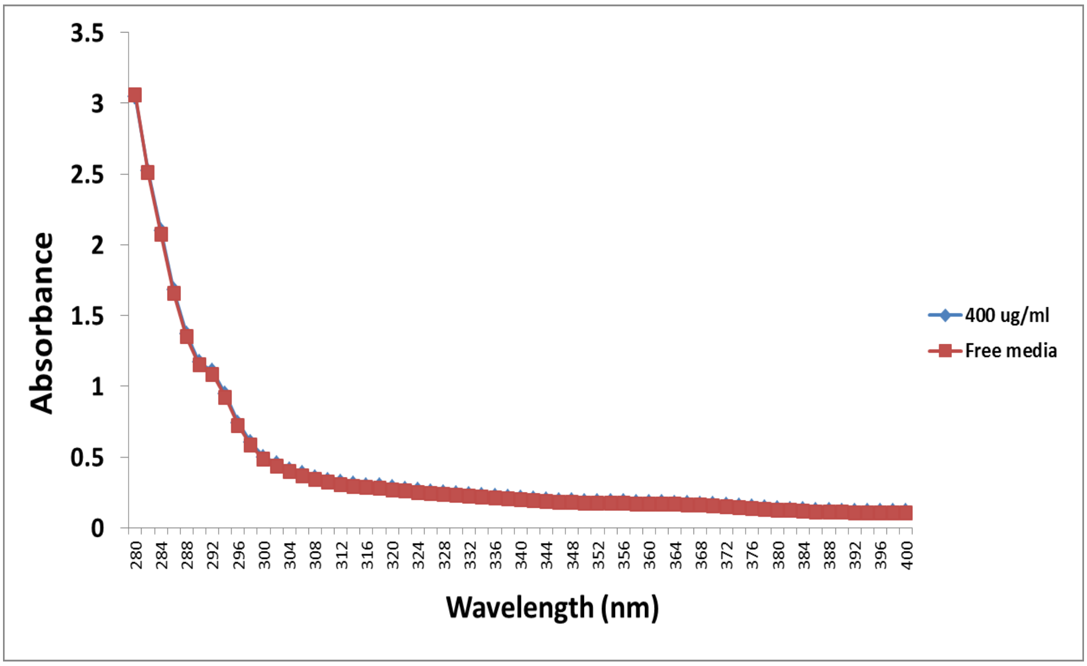

Figure S1. Relation between UV absorbance and wavelength (280 to 400 nm).
